# Supplementary material for: Elevated IL-1 Beta Plasma Levels, Altered Platelet Activation and Cardiac Remodeling Lead to Moderately Decreased LV Function in Alzheimer Transgenic Mice After Myocardial Ischemia and Reperfusion
Source: J Cardiovasc Dev Dis. 2026 Jan 26;13(2):64. doi: 10.3390/jcdd13020064 (PMC12941953; doi:10.3390/jcdd13020064)
Supplement: Supplementary file 1 [file jcdd-13-00064-s001.zip › jcdd-4050950-supplementary.pdf]

Supplementary Material

Supplementary Figures

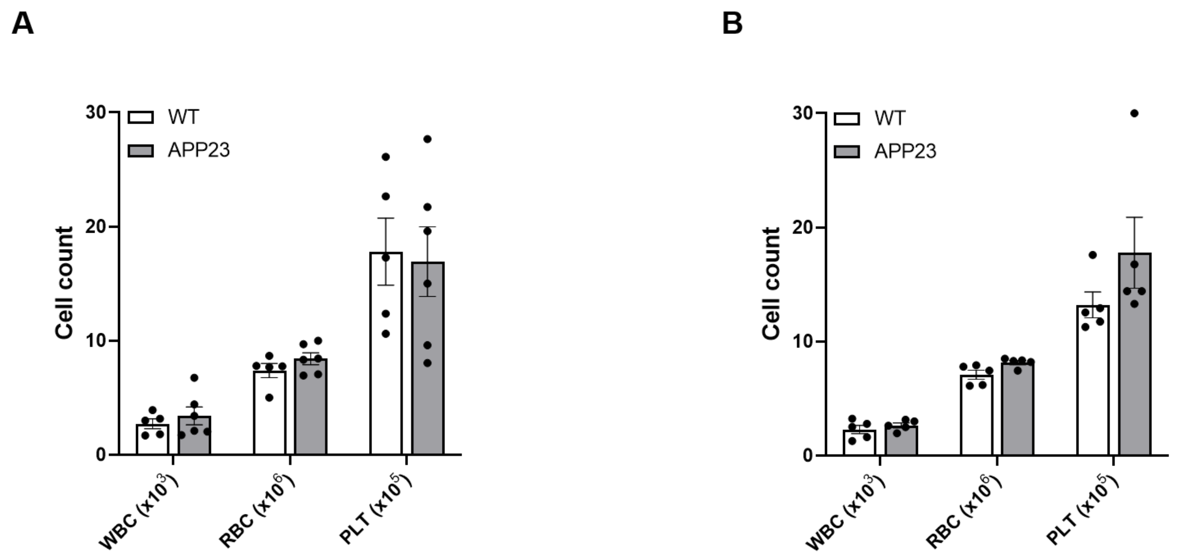

Supplementary Figure S1. Analysis of blood cell counts of WT and APP23 mice after AMI. (A-B) Unaltered white blood cell (WBC), red blood cell (RBC) and platelet (PLT) counts after 24h (A) and 21 days (B) of ischemia and reperfusion (n = 5). Bar graphs indicate mean values ± SEM. Statistical analyses were performed using a two-way ANOVA with Sidak's multiple comparison test.
